# An Ensemble Matrix Completion Model for Predicting Potential Drugs Against SARS-CoV-2

**DOI:** 10.3389/fmicb.2021.694534

**Published:** 2021-07-21

**Authors:** Wen Li, Shulin Wang, Junlin Xu

**Affiliations:** College of Computer Science and Electronic Engineering, Hunan University, Changsha, China

**Keywords:** virus-drug association prediction, matrix completion, SARS-CoV-2, weighted voting ensemble model, computational drug repurposing

## Abstract

Because of the catastrophic outbreak of global coronavirus disease 2019 (COVID-19) and its strong infectivity and possible persistence, computational repurposing of existing approved drugs will be a promising strategy that facilitates rapid clinical treatment decisions and provides reasonable justification for subsequent clinical trials and regulatory reviews. Since the effects of a small number of conditionally marketed vaccines need further clinical observation, there is still an urgent need to quickly and effectively repurpose potentially available drugs before the next disease peak. In this work, we have manually collected a set of experimentally confirmed virus-drug associations through the publicly published database and literature, consisting of 175 drugs and 95 viruses, as well as 933 virus-drug associations. Then, because the samples are extremely sparse and unbalanced, negative samples cannot be easily obtained. We have developed an ensemble model, EMC-Voting, based on matrix completion and weighted soft voting, a semi-supervised machine learning model for computational drug repurposing. Finally, we have evaluated the prediction performance of EMC-Voting by fivefold crossing-validation and compared it with other baseline classifiers and prediction models. The case study for the virus SARS-COV-2 included in the dataset demonstrates that our model achieves the outperforming AUPR value of 0.934 in virus-drug association’s prediction.

## Introduction

In 2019, a new type of respiratory disease caused by the virus SARS-CoV-2 had been officially named the coronavirus disease 2019 (COVID-19), whose outbreak caused a disaster for people around the world, affecting economics, politics, and life in every way. It can cause fevers, fatigue, cough, muscle soreness, and progressive breathing difficulties (Li et al., 2020). Also, it can infect alveolar epithelial cells, leading to severe bilateral peripheral pneumonia with glass opacity observed in the CT image, and cause severe acute respiratory distress syndrome (ARDS), hypoxia, and even death. So far, although the disease caused by SARS-CoV-2 has a low fatality rate, it is more contagious than SARS or MERS. As of June 3, 2021, there are currently 171,301,296 new coronavirus cases worldwide, resulting in 3,567,486 deaths. Despite the ongoing worldwide efforts to develop a vaccine against COVID-19, due to the limitations such as the high cost of low-temperature storage and transportation, lack of lasting immunity to evolving mutated viruses, and increasing antibody dependence, it is not easy to sustain long-term immunity from vaccines. It is expected that COVID-19 may be permanent and constantly mutating in the future, with multiple peaks of seasonal outbreaks before herd immunity occurs. Therefore, we need to spare no effort to find the right drugs against COVID-19. Computational drug repurposing is a promising strategy that can repurpose existing drugs whose safety, optimal dose, and pharmacokinetics are well known for immediate responses against COVID-19. In the sizeable virus-drug association space, it is not very practical to put all antiviral drugs into the clinical trials against COVID-19, especially in a time crunch. In this specific situation, computational models can reduce the searching space for trials and shorten preclinical approval costs. The drug computational repurposing task can also be remodeled as the association prediction task between viruses and drugs (Li et al., 2016; Luo and Long, 2020), which can be generally divided into three categories.

The first category is the network-based model (Zhang et al., 2018; Zeng et al., 2020), which usually constructs a heterogeneous interaction network and then performs all kinds of algorithms like network propagation or random walk on it (Luo and Xiao, 2017). For example, Long and Luo (2020) established a model for identifying microbe-drug associations based on heterogeneous networks, combined with embedding representation learning (HNERMDA). Unfortunately, this model of HNERMDA cannot predict associations for novel viruses (or drugs) because of the lack of links to neighbors. This type of network-based model heavily relies on the structure of the network and the algorithms running on it (Chen et al., 2012), so the complexity of the network topology will significantly influence the convergence speed of the method (Peng et al., 2018). Once an isolated node does not have any known association, the algorithm will get bogged down until restart (Wang et al., 2019; Zhang et al., 2019b). The second category is the machine learning–based method, which also can be divided into supervised learning and semi-supervised learning (Cai et al., 2018; Chen et al., 2018a). All the supervised learning methods such as Logistic Regression (LR), Random Forest (RF), Support Vector Machine (SVM) (Wang et al., 2007b), Gradient Boosting Decision Tree (GBDT), and Neural Network (NN) (Gao et al., 2021) require both positive and negative samples for training (Chen et al., 2018a). In contrast, the semi-supervised learning-based methods can train the model without negative samples. So, they are suitable for unbalanced or no negative sample scenarios with sparsely known associations (Luo et al., 2018; Liu et al., 2020; Chen Y. et al., 2021). Zhao et al. (2018) proposed a bipartite network projection recommendation algorithm and combined the lncRNA-protein association matrix, a protein similarity matrix, and a lncRNA similarity matrix to predict potential lncRNA-protein interaction. Zhang et al. (2019a) built an ensemble model of sparse feature learning with linear neighborhood regularization for drug-drug association prediction. The third category is the deep learning-based methods (Chen et al., 2019), which are particularly useful for large-scale data (Deng et al., 2020; Long et al., 2020a). Zeng et al. (2019) proposed a circular RNAs and disease association prediction model by deep forests that have solved the absence of negative samples. Long et al. (2020b) developed an ensemble model of graph attention networks to predict potential microbe-drug associations for human beings, which combines network structure and deep learning model. Yu et al. (2020) proposed a deep learning model for drug-disease association prediction combining layer attention graph convolutional network. These deep learning–based methods are effective, but the training time will increase as the search space expands.

Some hybrid models incorporate more than one approach (Wang et al., 2007a). For example, the prediction model proposed by Zhang et al. (2019c) integrated heterogeneous network and non-negative matrix factorization. In addition, Zhao et al. developed a novel model of lncRNA-miRNA association prediction by graphlet interaction and interactome network (Zhang et al., 2021). In order to effectively reduce the prediction variance and generalization error, improve the prediction accuracy, and make the performance exceed that of a single model, ensemble multiple models through appropriate combination strategies (such as RF, boosting, stacking, voting) has been widely recognized as an effective method. In this paper, to take advantages of different types of matrix completion methods, we developed an ensemble model based on matrix completion and weighted soft voting (called EMC-Voting) to predict potentially associated drugs for SARS-CoV-2. The soft voting model is a weighted ensemble model based on the assumption that the higher the base model’s performance, the more significant its contribution to the final result. Therefore, we estimate the voting weight of each model by its proportion of AUPR value. The experimental results showed that EMC-Voting demonstrated superior prediction performance in fivefold crossing validation, achieving an average AUC of 93.24%, average AUPR value of 91.74%, and average accuracy of 87.61%.

**FIGURE 1:**
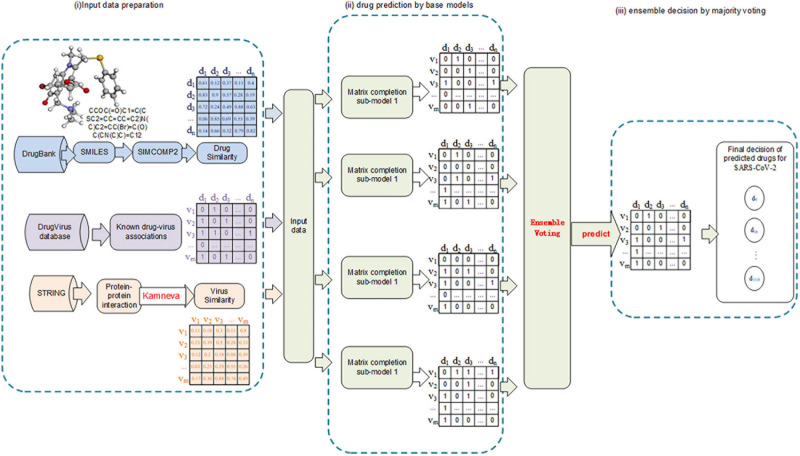
Flowchart of our work.

The workflow of this study mainly involves the following: (i) Collect and summarize the human virus-drug associations from public databases and calculate the integrated similarity for viruses and drugs, respectively. (ii) Test multiple prediction models, including machine learning–based models (supervised), matrix completion models (semi-supervised), and deep learning models, and select the ones with the best performance. (iii) Ensemble the selected models by the weighted soft voting strategy and predict a probability score list for all the virus-drug pairs. The flowchart of our model can be seen in Figure 1.

## Materials and Methods

### Problem Description

There are numerous studies and experiments to confirm the associations between viruses and broad-spectrum antiviral drugs. According to the hypothesis, similar viruses tend to interact with similar drugs. This study attempts to predict potential virus-drug associations, especially those most related to the virus SARS-CoV-2, by combining known virus-drug association, virus-virus similarity, and drug-drug similarity. The existing virus-drug associations are extracted from public published databases. We construct a virus-drug adjacency matrix to represent binary interaction information. Virus similarity is integrated by virus function similarity and Gaussian similarity, and drug similarity is integrated by chemical structure similarity and Gaussian similarity.

The virus-drug association prediction problem can be modeled as a classification problem. By predicting, a list of scores corresponding to all the virus-drug pairs is obtained. Then, the virus-drug pairs are prioritized according to the predicted scores. Moreover, they are ranked to infer the potential associations between viruses and drugs. We use an adjacency matrix *A* ∈ ℜ^*m*×*n*^ to represent the known virus-drug associations, with rows representing viruses and columns representing drugs. If there is an association between virus *v*_*i*_ (1≤*i*≤*m*) and drug *d*_*j*_ (1≤*j*≤*n*), the element *A*(*i*,*j*) in the adjacency matrix will be assigned 1, or else 0. We denoted the symbol Ω as a set of indices of partially known entries in the matrix*A*. The symbol *P*_Ω_(*A*) is defined as a linear projection operator subject to the Equation 1:

(1) $$P_{\Omega }\,{\left(A\right)}_{i\,j}=\left\{ \begin{array}{c} A_{i\,j},i\,f\,\left(i,j\right)\in \Omega \\ 0,i\,f\,\left(i,j\right)\in \Omega \end{array}\right.$$

### Data Collection and Preprocessing

#### The Known Virus-Drug Associations

The public database DrugVirus^1^ (Andersen et al., 2020) documents the development of 118 compounds against 83 human viruses, including the status of drug development against virus SARS-CoV-2. These compounds (drugs) are broad-spectrum antiviral agents (BSAAs) that target viruses belonging to two or more virus families. In addition, we updated several experimentally confirmed virus-drug associations through relevant drug databases and published literature, including other 12 viruses and 57 drugs. As recorded in Table 1, 933 virus-drug associations are obtained from 95 viruses and 175 drugs. We utilized an adjacency matrix *A* to denote the known virus-drug associations.

**TABLE 1:** Statistics of the dataset.

**Dataset**	**#Viruses**	**#Drugs**	**#Associations**	**Sparsity**
DrugVirus	95	175	933	94.39%

*“#” represents “the number of.”*

It is important to note that there is no clinically or experimentally confirmed drug for COVID-19 in the world. However, many drugs have been found to have specific inhibitory and mitigating effects *in vivo* or *in vitro*. As a result, a few antiviral drugs are being focused on and put into clinical trials with the hope of being marketed and approved. In the DrugVirus database, drugs such as Cepharanthine, Chloroquine, Homoharringtonine, Hydroxychloroquine, Ivermectin, Lopinavir, Mefloquine, Remdesivir, and Ribavirin are recorded in the stage of cell cultures/co-cultures, and Arbidol (or named Umifenovir) and Hydroxychloroquine are recorded in the clinical phase III (Andersen et al., 2020). Therefore, to predict more than just these 10 drugs, the associations between SARS-CoV-2 and these drugs are considered known (the corresponding virus-drug pairs are assigned value 1 in the adjacency matrix). We planned to develop an ensemble model to predict potential SARS-CoV-2-associated drugs, which can help clinicians select effective therapeutic agents or help researchers identify appropriate drugs for subsequent clinical trials.

#### Virus Similarity

We calculate the virus functional similarity as that in reference (Long et al., 2020a). First, we extracted protein-protein interactions (PPI) from the database STRING^2^ by the virus names. Then, we constructed a protein-protein functional network. Hence, the functional similarity of virus-virus in the virus functional network is calculated by the method proposed by Kamneva (2017). At last, we used a matrix *FS* ∈ *ℝ*^*m*×*m*^ to represent the virus function similarity network. The value of the matrix element *FS*(*v*_*i*_,*v*_*j*_) represents the similarity score between virus *v*_*i*_ and virus *v*_*j*_. Since not every pair of proteins has functional interaction, many pairs of viruses have no functional similarity. Thus, the matrix *FS* is extremely sparse, with many elements having a value of 0. In order to measure the virus-virus similarity more comprehensively, we calculated the Gaussian interaction profile (GIP) kernel similarity *GIP*_*v*_ ∈ *ℝ*^*m*×*m*^ for all the virus-virus pairs by leveraging the known virus-drug association. The GIP kernel similarity *GIP*_*v*_(*v*_*i*_,*v*_*j*_) between *v*_*i*_ and *v*_*j*_ can be calculated as the Equation 2.

(2) $$G\,I\,P_{v}\,\left(v_{i},v_{j}\right)=\exp\left(-\gamma_{v}\,{||I\,P\,\left(v_{i}\right)-I\,P\,\left(v_{j}\right)||}^{2}\right)$$

where ||⋅|| denotes the norm of a vector. The symbol γ_*v*_ is the normalized kernel bandwidth: $\gamma_{v}=\gamma_{v}^{\prime }\,/\,\left(\frac{1}{m}\,\sum_{i=1}^{m}{||I\,P\,\left(v_{i}\right)||}^{2}\right)$. $\gamma_{v}^{\prime }$ is the original bandwidth and usually set to 1. The binary vector *IP*(*v*_*i*_) is the i-th row of the association matrix *A*, denoting whether there is an association between *v*_*i*_ and all the drugs. As a result, based on the above functional similarity *FS*(*v*_*i*_,*v*_*j*_) and Gaussian kernel similarity *GIP*_*v*_(*v*_*i*_,*v*_*j*_), the integrated virus similarity *S*_*v*_(*v*_*i*_,*v*_*j*_) between virus *v*_*i*_ and virus *v*_*j*_ is calculated as Equation 3:

(3) $$S_{v}\left(v_{i},v_{j}\right)=\{\begin{array}{cc} \frac{FS\left(v_{i},v_{j}\right)\;+\;GIP_{v}\left(v_{i},v_{j}\right)}{2}, & \text{if }FS\left(v_{i},v_{j}\right)\neq 0\\ GIP_{v}\left(v_{i},v_{j}\right), & \text{otherwise} \end{array}$$

#### Drug Similarity

We calculated the drug chemical structure similarity by the method of SIMCOM2 (Hattori et al., 2010). First, we get the compound IDs (Entry number) of the drugs from the database KEGG^3^ by the names of the drugs. Then, we input the IDs of all drugs into SIMCOMP2, which can calculate the structural similarity score between all drug-drug pairs in batches based on their structural information obtained by IDs. Finally, we denoted the structural similarity of all the drug-drug pairs as used as matrix *DS* ∈ *ℝ*^*n*×*n*^. The value of the element *DS*(*d*_*i*_,*d*_*j*_) represents the similarity score between drug *d*_*i*_ and drug *d*_*j*_.

Like the virus interaction vector *IP*(*v*_*i*_), the drug interaction vector *IP*(*d*_*j*_) is the column vector of the association matrix *A*. The GIP similarity between *d*_*i*_ and *d*_*j*_, denoted as *GIP*_*d*_(*d*_*i*_,*d*_*j*_), also can be calculated by substituting the *IP*(*d*_*i*_), *IP*(*d*_*j*_), and γ_*d*_ into Equation 2. As a result, the integrated drug similarity *S*_*d*_(*d*_*i*_,*d*_*j*_) between drug *d*_*i*_ and drug *d*_*j*_ is calculated as Equation 4:

(4) $$S_{d}\left(d_{i},d_{j}\right)=\{\begin{array}{cc} \frac{DS\left(d_{i},d_{j}\right)\;+\;GIP_{d}\left(d_{i},d_{j}\right)}{2}, & \text{if }DS\left(d_{i},d_{j}\right)\neq 0\\ GIP_{d}\left(d_{i},d_{j}\right), & \text{otherwise} \end{array}$$

According to the hypothesis, similar viruses will tend to be associated with similar drugs. Therefore, appropriate similarity measures will help predict potential virus-drug associations. In this study, we used the final integrated virus (drug) similarity [*S*_*d*_(*d*_*i*_,*d*_*j*_)].

### Base Model Selection

Since the number of known drug-virus associations is far less than the total number of virus-drug pairs, the virus-drug association adjacency matrix is highly sparse. Moreover, the virus-drug association matrix is highly unbalanced and biased due to the lack of negative samples (negative sample cannot be easily determined), just as with positive samples and unlabeled samples. For all these reasons, traditional supervised machine learning classifiers that both need positive labels and negative labels are not suitable for predicting potential virus-drug associations. Similarly, the deep learning model, which requires a large number of samples for training, is not suitable for this prediction scenario. Thus, this study takes advantage of the matrix completion method for prediction, a semi-supervised machine learning model that does not require negative samples and has made remarkable achievements in the recommender system. Most importantly, the method of matrix completion can predict the drugs associated with a *de novo* virus (one without any known association) and vice versa.

Various matrix completion (MC) models have been developed for association prediction and work well. However, each of them has its limitations and shortcomings. In order to overcome the shortcoming of one single model, ensemble learning is considered an effective method. Ensemble learning is a kind of hybrid model whose prediction is accurate and robust. There are also many ensemble strategies such as voting, RF, boosting, and stacking. In our study, weighted soft voting is utilized to ensemble multiple matrix completion models to predict potential associated drugs against the virus SARS-CoV-2. We fitted the known virus-drug associations, virus integrated similarities, and drug integrated similarities into seven matrix completion models, including the model of Graph Regularized Matrix Factorization (GRMF) (Ezzat et al., 2017), Inductive Matrix Completion (IMC) (Chen et al., 2018b), Bounded Nuclear Norm Regularization (BNNR) (Yang et al., 2019), Faster Randomized Matrix Completion (FRMC) (Li et al., 2019), Deep Matrix Factorization (DMF) (Fan and Cheng, 2018), Deep Learning-based Matrix Completion (DLMC) (Fan and Chow, 2017), and Heterogeneous Graph Inference with Matrix Completion (HGIMC) (Yang et al., 2021). All of these models have performed well in the field of biological association prediction. We selected and tested different combinations of these base models by fivefold cross-validation to develop an ensemble model. The combination of model GRMF, FRMC, BNNR, and HGIMC were selected to build our Ensemble Matrix Completion-based Voting (EMC-Voting) model, which achieved the highest AUPR value.

As discussed above, the association adjacency matrix is an incomplete binary matrix, so the purpose of matrix completion is to retrieve the missing items in the adjacency matrix *A*, assuming that the entries in the recovered matrix should be as close as possible to entries in the original adjacency matrix (Ramlatchan et al., 2018). Combined with iterative optimization techniques, the matrix completion model is efficient and fast (Chen X. et al., 2021). Furthermore, due to the assumption that the underlying determinants of the virus-drug association are closely related, the number of independent elements is less than the number of drugs or viruses. Therefore, the virus-drug association matrix to be recovered is low-rank or approximately low-rank. So, the recovery of the matrix *A* can be resolved by constructing a low-rank approximate matrix *A*^∗^ that satisfies the equation: $A_{i\,j}^{*}=A_{i\,j}$ for all elements (*i*,*j*) ∈ Ω (Ω is the set of indices partially observed in the matrix *A*). The four basic matrix completion models selected and ensembled in our EMC-Voting model are described below.

#### Graph Regularized Matrix Factorization Model

Graph Regularized Matrix Factorization (GRMF), one of the mathematical models of matrix completion, is proposed by Ezzat et al. (2017). The model of GRMF decomposes the virus-drug association adjacency matrix *A* ∈ *ℝ*^*m*×*n*^ into two low-rank feature matrices *L*_*f*_ ∈ *ℝ*^*m*×*k*^ and *R*_*f*_ ∈ *ℝ*^*k*×*n*^ for viruses and drugs, respectively, based on the principle of low-rank approximation (LRA). *k* is the number of the latent independent features in the feature matrix *L*_*f*_ and *R*_*f*_. The objective function of GRMF is $\min_{L_{f},R_{f}}{||A^{*}-L_{f}\,R_{f}^{T}||}_{F}^{2}$. To prevent overfitting, graph regularization terms and Tikhonov are added, and the objective function is converted as Equation 5:

(5) $$\min_{L_{f},R_{f}}{||A^{*}-L_{f}\,R_{f}^{T}||}_{F}^{2}+\lambda_{l}\,\left({||L_{f}||}_{F}^{2}+{||R_{f}||}_{F}^{2}\right)+\lambda_{v}\sum_{i,r=1}^{m}{\hat{N}}_{i\,r}^{v}\,{||a_{i}-a_{r}||}^{2}+\lambda_{d}\,\sum_{j,q=1}^{n}{\hat{N}}_{j\,q}^{d}\,{||b_{j}-b_{q}||}^{2}$$

where λ_*l*_, λ_*v*_, λ_*d*_ are the balance parameters, *a*_*i*_, *b*_*j*_ are the i-th and the j-th row of the matrix *L*_*f*_ and *R*_*f*_, respectively. ${\hat{N}}^{v}$ and ${\hat{N}}^{d}$ are p-nearest neighbor graph derived from virus and drug similarity matrices, respectively. The Frobenius norm *L*_*f*_ and *R*_*f*_ are minimized by Tikhonov regularization (Gu et al., 2010).

By fivefold CV, hyperparameters of GRMF are tuned using grid search. The parameter *k* is tuned in the range of [50, 95], λ_*l*_ in the range of {2^−2^,2^−1^,2^0^,2^1^}, λ_*v*_/λ_*d*_ in the range of {0,10^−4^,10^−3^,10^−2^,10^−1^}, and ${\hat{N}}^{v}$/${\hat{N}}^{d}${1,2,3,4,5,6,7,8}. All the parameters are tuned and determined as listed in Table 2.

**TABLE 2:** Parameters setting.

**Base model**	**Parameters**
GRMF	lambda_l = 2, lambda_d = 0.1, lambda_t = 0.1, *k* = 85, *p* = 5, num_iter = 2
BNNR	alpha = 10, beta = 10
FRMC	tol = 0.1, i_reuse = 50, q_reuse = 10, ran = [0,1]
HGIMC	alpha = 10, beta = 10, gamma = 0.7, threshold = 0.5, sigma = 0.5

#### Faster and Randomized Matrix Completion Model

Fast Randomized Matrix Completion (FRMC) is a computational model that has been used to predict the latent associations between lncRNAs and diseases (Li et al., 2019). It is a type of rank minimization model. In this model, the augmented matrix *M* ∈ *ℝ*^(*m* + *n*)×(*m* + *n*)^ represents the adjacency matrix of the bilayer heterogeneous virus-drug association network, defined as: $\text{M}=\left[\begin{array}{cc} S_{v} & A\\ A^{T} & S_{d} \end{array}\right]$, where *S*_*v*_ and *S*_*d*_ are the similarity matrix of viruses and drugs. *A* ∈ *ℝ*^*m*×*n*^ is the adjacency matrix of virus-drug association matrix, and *A*^*T*^ is the transpose matrix of *A*. The task of matrix completion for matrix *M* can be formulated as a problem of rank minimization. The objective function is defined as:

(6) $$\min rank\left(R\right)\;s.t.,P_{\Omega }\left(R\right)=P_{\Omega }\left(M\right)$$

where *R* is the approximate matrix (the predicted/recovered matrix), *rank*(*R*) is the rank of *R*. Unfortunately, rank minimization is an NP-hard problem (Candès and Recht, 2009). Thus, the problem of rank minimization is then relaxed to the nuclear normal minimization problem formulated as:

(7) $$\min\tau ||R|{|}_{{}^{*}}+\frac{1}{2}||R|{|}_{F}^{2}\;s.t.,P_{\Omega }\left(R\right)=P_{\Omega }\left(M\right)$$

where ||⋅||_*_ is the nuclear norm, ||⋅||_*F*_ is the Frobenius norm, τ is thresholding (usually set to ${||{\text{P}}_{\Omega }\,\left(M\right)||}_{F}\,\left(m+n\right)/\sqrt{\left|\Omega \right|}$). The problem of nuclear norm minimization can be resolved by the Singular Value Thresholding (SVT) algorithm with linear Bregman iterations (Hastie et al., 2015). The error tolerance *tol* controls the condition for the end of the iteration. Parameters i_reuse and q_reuse are the numbers of two kinds of reuses. They are all tuned in our ensemble model, as shown in Table 2.

#### Bounded Nuclear Norm Regularization Matrix Completion Model

Under the low-rank hypothesis, BNNR has been used to retrieve the drug-disease association matrix (Yang et al., 2019). To deal with noisy data, the condition of constraint is added to a regularization model. The optimization model of nuclear norm minimization can be resolved as:

(8) $$\min_{S}{||R||}_{{}^{*}}+\frac{\alpha }{2}\,{||{\text{P}}_{\Omega }\,\left(R\right)-{\text{P}}_{\Omega }\,\left(M\right)||}_{F}^{2}\,\;s.t\,.0\leq R\leq 1$$

where the matrix *R* is the predicted matrix (recovered matrix). *P*_Ω_(⋅) is a linear projection operator, the parameter α is used to balance the nuclear norm and the error. The model of BNNR can be solved by the Alternating Direction Method of Multipliers (ADMM) (He et al., 2000). There are two hyperparameters, alpha and beta, that need to be tuned in the range {0.1,1,10,100}. The final values of these two parameters are also listed in Table 2.

#### Heterogeneous Graph Inference With Matrix Completion Model

The model of heterogeneous graph inference with matrix completion (HGIMC) achieves matrix completion at three steps (Yang et al., 2021): Firstly, the Boundary Matrix Completion (BMC) model is used to prepopulate some of the missing entries in the original virus-drug association matrix. Secondly, Gaussian Radial Basis Function (GRB) improves the similarity of virus and drugs. Then, a new heterogeneous virus-drug network is constructed based on the updated virus-drug association and the new similarity. Thirdly, HGIMC uses heterogeneous networks to infer unknown virus-drug pairs scores and recommend promising indications for a virus. The hyperparameters in the original paper are also tuned and decided in Table 2.

#### Model Ensembling

The parameters in the model of EMC-Voting were tuned on the training set by fivefold CVs using grid search. As shown in Table 2, they were finally decided according to the highest AUPR values. Our EMC-Voting model is ensembled and implemented by these four selected models, GRMF, BNNR, FRMC, and HGIMC, by a weighted soft voting strategy, based on the assumption that the better the performance, the greater the contribution. At last, the weights of Ensembling were finalized by the proportion of their AUPR values.

## Experiments and Results

In this section, we first introduced the performance evaluation metrics adopted in our model. Then, we compared our EMC-Voting model with several machine learning-based models and single MC-based models by fivefold CV on the same dataset to evaluate the predictive performance of our model. Furthermore, to test the prediction ability of EMC-Voting for the *de novo* virus, we deleted all known associations of the virus SARS-CoV-2 and used our model to predict associated drugs to see if the known associations were correctly predicted. At last, we conducted case studies on the top 40 predictive drugs against the virus SARS-COV-2. In our experiments, all the matrix completion models, including our EMC-Voting model, are conducted in the MATLAB 2017b, the operating system of windows10, with AMD Ryzen 5 3600 6-core, 3.59 GHz CPU, 32G memory. Furthermore, all the machine learning and deep learning comparison models are run on the system of python 3.7 with TensorFlow 2.2.0.

### The Evaluation Metrics for Prediction Performance

First, in order to make a fair comparison with machine learning classifiers (which require negative samples) and uniformly evaluate their prediction performance, we constructed negative samples with the same size as positive samples by the method proposed by Zhao et al. (2019). Before sampling, all the unlabeled samples were divided into 23 clusters. Then, from each cluster, negative samples with the same number of positive samples were randomly sampled. In our fivefold cross-validation experiments, all the labeled samples (including positive and negative) were randomly divided into five non-overlapping subsets, and each subset was taken in turn as the test sample in each fold cross-validation other four subsets were taken as the training sample. The execution repeated until all samples have been tested once, and one round fivefold CV ends. Since the test set and training set are randomly divided each time, we run 20 rounds of cross-validation and take the average at the end. In this study, we employed the following eight metrics, Accuracy, F1 scores, Sensitivity, Specificity, PPV, NPV, AUC, and AUPR, to demonstrate the performance of our model and other models. The confused matrix counts four important indicators according to the predicted label and the original label: true positive (TP), true negative (TN), false positive (FP), and false-negative (FN). Therefore, all eight metrics can be calculated from these four indicators. Accuracy, the proportion of correctly classified samples (TP+TF) to the total number of samples, can be calculated as:

(9) $$A\,c\,c\,u\,r\,a\,c\,y=\frac{T\,P+T\,N}{T\,P+T\,N+F\,P+F\,N}\times 100\%$$

F1 score can be interpreted as a weighted average of accuracy and recall rates:

(10) $$F\,1=\frac{2\times \text{precision}\times \text{recall}}{\text{precision}+\text{recall}}$$

**FIGURE 2:**
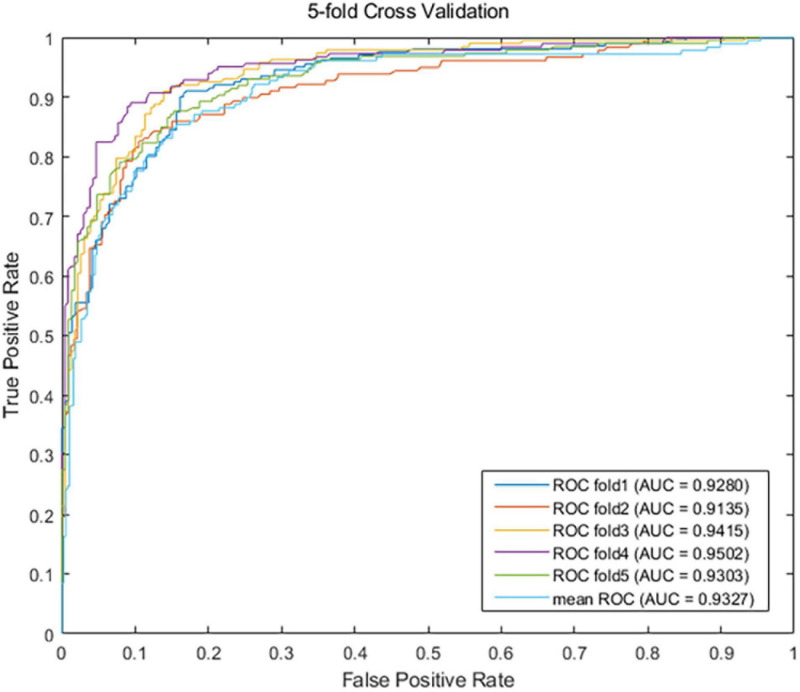
The ROC curve of EMC-voting by one time fivefold cross-validation.

Sensitivity is the proportion of true positive: *Sensitivity* = *TP*/(*TP* + *FN*) = *Recall*. Specificity is the proportion of true negative: *Specificity* = *TN*/(*TN* + *FP*). PPV is the positive predictive value, which is equal to Precision: *PPV* = *TP*/(*TP* + *FP*) = Precision. NPV is the negative predictive value: *NPV* = *TN*/(*TN* + *FN*). AUC is the area under the ROC curve. Importing *metrics* from the *sklearn* library, we called the function *roc_curve* to draw the ROC curve, and the function *auc* is called to calculate the AUC value. AUPR is the area under the precision-recall (PR) curve. The function *precision*_*recall*_*curve* is called to calculate the Precision value and the recall value, and the function *auc* also can be called to calculate the AUPR value. The greater the metrics values are, the better the predictive performance is. So, the value of AUPR is employed as the main metric for performance evaluation. After one round fivefold CV, our EMC-Voting model achieves AUC values of 0.9280, 0.9135, 0.9415, 0.9502, 0.9303, and 0.9327 at each fold, respectively. The mean value of AUC of one round fivefold cross-validation is 0.9327. As shown in Figure 2, the results demonstrate that the EMC-Voting model can effectively predict potential virus-drug associations on this dataset.

### Comparison of Prediction Performance

#### Compare With Machine Learning–Based Models

First, we compared our model with SVM, GBDT, NN, ERLX, and SPMCIIP models. The classifiers of SVM, GBDT, and NN were called with the method of “SVC,” “lightgbm,” and “mlp,” respectively. The model SPMCIIP ensembled SVM, GBDT, and NN classifiers with weights “0.3, 0.5, 0.2” to predict critical COVID-19 (Gao et al., 2021). The model of ERLX ensembles three diverse classifiers: Extra Trees, RF, and LR by Extreme Gradient Boosting (XGBoost) to achieve the ensembling (AlJame et al., 2020). The parameters of all comparison methods are the same as the original paper. All the models run on the same dataset, with the same test set and the same training set. After 20 times fivefold CVs, the average results are shown in Table 3. It can be found that the AUPR values of SVM, GBDT, MLP, SPMCIIP, and ERLX are 0.8255, 0.9078, 0.8727, 0.8951, 0.9091, and 0.9174, respectively. Except for the metrics of specificity and PPV, all the metrics of the EMC-Voting model are better than those of other classifiers and models, achieving the highest performance.

**TABLE 3:** Performance comparison between our EMC-voting and machine learning-based classifier.

**Classifiers**	**Accuracy**	**F1**	**Sensitivity**	**Specificity**	**PPV**	**NPV**	**AUPR**	**AUROC**
SVM	0.8033 ± 0.0044	0.7866 ± 0.0044	0.8094 ± 0.0059	0.7983 ± 0.0075	0.766 ± 0.0065	0.8382 ± 0.0042	0.8255 ± 0.004	0.8693 ± 0.0022
GBDT	0.8559 ± 0.0035	0.8364 ± 0.0048	0.8229 ± 0.009	0.8827 ± 0.0032	0.8511 ± 0.0029	0.8605 ± 0.0059	0.9078 ± 0.0023	0.9299 ± 0.0017
MLP	0.8513 ± 0.0032	0.8308 ± 0.0038	0.816 ± 0.005	0.8799 ± 0.0037	0.8469 ± 0.0044	0.8554 ± 0.0035	0.8727 ± 0.0029	0.8479 ± 0.0033
SPMCIIP	0.8544 ± 0.002	0.8355 ± 0.0023	0.826 ± 0.0037	0.8776 ± 0.0032	0.846 ± 0.0033	0.8618 ± 0.0025	0.8951 ± 0.0021	0.9191 ± 0.0015
ERLX	0.8705 ± 0.003275	0.8586 ± 0.003825	0.8542 ± 0.0059	0.8844 ± 0.0044	0.8632 ± 0.004275	0.8767 ± 0.0040	0.9091 ± 0.0028	0.8923 ± 0.002175
**EMC-Voting**	**0.8761 ± 0.0008**	**0.8641 ± 0.0007**	**0.8791 ± 0.0014**	0.8739 ± 0.0020	0.8503 ± 0.002	**0.8988 ± 0.0010**	**0.9174 ± 0.0001**	**0.9324 ± 0.0001**

*Bold values are the best performance values.*

#### Compare With the State-of-the-Art Matrix Completion Models

To further test the prediction performance for the virus-drug associations, we compare our EMC-Voting model with the state-of-the-art matrix completion-based models by fivefold CV on the virus-drug dataset. IMCMDA was first proposed by Chen et al. (2018b) which used inductive matrix completion to predict potential microRNA-disease associations, achieving good performance. DLMC is a deep learning-based matrix completion method proposed by Ran et al., which utilizes autoencoder based matrix completion (AEMC) to do the pre-training step for both the missing entries and network parameters (Fan and Chow, 2017). DMF is a model of non-linear matrix completion constructed as a deep-structure NN proposed by Fan and Cheng (2018) for image inpainting and collaborative filtering. DMF can simultaneously optimize the inputs and the parameters of the model to reduce errors, which is suitable for large matrices with high accuracy.

**FIGURE 3:**
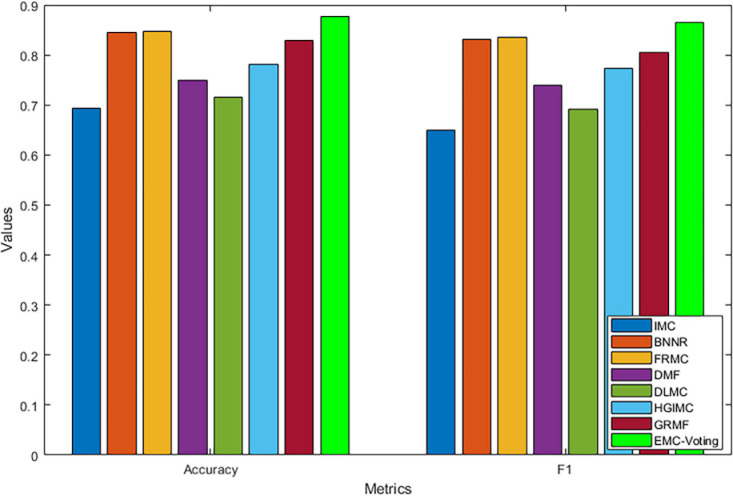
Comparison of performance of different prediction models by Accuracy and F1 scores.

The results of our model and all the comparison models are shown in Table 4 and Figure 3. EMC-Voting obtains the highest value of AUPR, which is at least 3.03% higher than the other seven models. Furthermore, EMC-Voting outperforms the other seven models according to all the metrics of the results.

**TABLE 4:** Comparison between the EMC-voting model and other matrix completion-based models.

**Method**	**Accuracy**	**F1**	**Sensitivity**	**Specificity**	**PPV**	**NPV**	**AUPR**	**AUROC**
IMCMDA	0.6937	0.6505	0.6399	0.7026	0.7205	0.7104	0.7092	0.6576
BNNR	0.8454	0.8318	0.8616	0.8306	0.8045	0.8815	0.8904	0.9059
FRMC	0.8477	0.8345	0.8629	0.8324	0.8094	0.8836	0.8870	0.9039
DMF	0.7489	0.7390	0.7930	0.7055	0.6941	0.8070	0.7912	0.7935
DLMC	0.7153	0.6906	0.7119	0.7203	0.6741	0.7532	0.7047	0.7616
HGIMC	0.7816	0.7725	0.8327	0.7342	0.7287	0.8530	0.8306	0.8599
GRMF	0.8286	0.8046	0.7939	0.8508	0.8174	0.8386	0.8687	0.8704
EMC-voting	**0.8761**	**0.8641**	**0.8791**	**0.8739**	**0.8503**	**0.8988**	**0.9174**	**0.9324**

*Bold values are the best performance values.*

**FIGURE 4:**
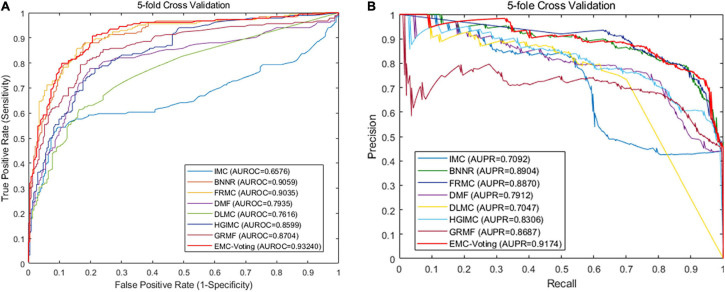
The results by 20 times fivefold CV. **(A)** ROC curve. **(B)** Precision-Recall curve.

Although DMF and DLMC are deep learning–based methods that perform well in text recognition, they do not perform excellently on this DrugVirus dataset. Probably because the size of our dataset is small and the number of samples is not enough for adequate training. Figure 4 shows the receiver operator characteristic (ROC) curve and PR curves of all these matrix completion-based comparison models. It can be found that the average AUC and AUPR values of EMC-Voting are the highest, and the curve trajectory is the best. In conclusion, EMC-Voting has the best performance in predicting potential virus-drug associations. That is because it employs a semi-supervised matrix completion approach and ensembles several good performing models through simple and effective weights.

### Predict Potential Associated Drugs for *de novo* Virus

COVID-19 is a new disease, and there is currently no experimentally confirmed treatment for it. On the public database DrugVirus, 10 drugs under clinical studies have been considered to have great potential for subsequent clinical trials, such as Remdesivir, Ribavirin, Arbidol\Umifenovir, Cepharanthine, Chloroquine, Homoharringtonine, Hydroxychloroquine, Ivermectin, Lopinavir, and Mefloquine. To simulate a *de novo* virus, we deleted all associations between the virus SARS-CoV-2 and its associated drugs. After predicting through our EMC-voting model, the top 30 predicted drugs were compared with the list of drugs published in the database DrugVirus. Results are demonstrated in Table 5. Seven out of 10 drugs in the DrugVirus are correctly predicted by our EMC-Voting model, suggesting that our EMC-Voting model can predict reliable and effective associated drugs for *de novo* virus, consistent with the advantages of the matrix completion algorithm.

**TABLE 5:** The TOP 30 predicted drugs associated with the *de novo* virus SARS-COV-2.

**Rank**	**Drugs**	**Confirm**	**Rank**	**Drugs**	**Confirm**	**Rank**	**Drugs**	**Confirm**
1	Ribavirin	Yes	11	Monensin	No	21	Emetine	No
2	Favipiravir	no	12	Silvestrol	No	22	Chloroquine	Yes
3	Remdesivir	Yes	13	Nitazoxanide	No	23	Homoharringtonine	Yes
4	Mycophenolic acid	No	14	Vidarabine	No	24	Lamivudine	No
5	Pleconaril	No	15	Obatoclax	No	25	Lopinavir	Yes
6	Cidofovir	No	16	ABT-263	No	26	Ivermectin	Yes
7	EIPA (amiloride)	No	17	Arbidol (Umifenovir)	Yes	27	Indinavir	No
8	Brequinar	No	18	Hexachlorophene	No	28	Artesunate	No
9	Itraconazole	No	19	Brincidofovir	No	29	Tilorone (Amixin)	No
10	BCX4430 (Galidesivir)	No	20	Zanamivir	no	30	Pentosan polysulfate	No

### Case Study

Finally, we leveraged our EMC-Voting model to predict potentially therapeutic drugs against the virus SARS-COV-2 and implemented a case study on the TOP 15 predicted drugs against SARS-COV-2. Although there are no experimentally confirmed drugs against SARS-COV-2, a few drugs such as Arbidol\Umifenovir, Cepharanthine, Chloroquine, Homoharringtonine, Hydroxychloroquine, Ivermectin, Lopinavir, Mefloquine, Remdesivir, and Ribavirin are already in clinical trials and are recorded as associated with the SARS-COV-2. All these associations published by the database DrugVirus were considered known and input into our ensemble model for prediction. After predicting, we obtained a predictive score matrix for all virus-drug pairs. Then, we deleted all known associated drugs for each virus (row in the score matrix) and sorted the predicted drugs in descending order. As a result, the TOP 15 predicted drugs for SARS-COV-2 are demonstrated in Table 6.

**TABLE 6:** Top 15 predicted drugs potentially associatedwith SARS-CoV-2.

**Rank**	**Drug_name**	**Evidence**	**Rank**	**Drug_name**	**Evidence**
1	Favipiravir	Cai et al., 2020	9	Nelfinavir	NCT04471662, 2020
2	ABT-263	No	10	Brequinar	NCT04575038, 2020
3	Emetine	Choy et al., 2020	11	Nitazoxanide	Calderón et al., 2020
4	Silvestrol	Mani et al., 2020	12	Obatoclax	Varghese et al., 2021
5	Amodiaquine	Weston et al., 2020	13	EIPA (amiloride)	Varghese et al., 2021
6	Chlorpromazine	Xu et al., 2020	14	BCX4430 (Galidesivir)	Sareen et al., 2020
7	Luteolin	No	15	Niclosamide	Weiss et al., 2021
8	Mycophenolic acid	Han et al., 2021			

It can be seen that most of the predicted drugs by the EMC-Voting model have been successfully verified in current clinical treatments. For example, the antiviral efficacy of Favipiravir was evaluated using a Syrian hamster model. Favipiravir was found to have a strong dose effect when treatment was initiated before or at the same time as infection, leading to decreased titers of pulmonary infection and clinical relief of COVID-19 (Driouich et al., 2021). Weiss et al. (2021) verified the antiviral efficacy of Niccloamide in a human airway model of SARS-CoV-2 and found Niccloamide is effective against D614G, B.1.1.7, and B.1.351 varieties. Their experiments highlighted their great potential as a therapeutic agent for COVID-19. BCX4430 (Galidesivir) is a broad-spectrum antiviral drug that has potential in the treatment of COVID-19. It is safe and well-tolerated in phase I trials. Galidesivir is currently undergoing phase II human trials of coronavirus in Brazil and worldwide (Sareen et al., 2020). Some predicted drugs are supported by other literature, and the evidence is shown in Table 6. Therefore, our EMC-Voting model can predict reliable and effective drugs for the virus SARS-COV-2.

## Conclusion and Discussion

The main contributions of this study are the following: (1) We have proposed a weighted soft voting ensemble model perceiving the accuracy of different matrix completion models. (2) Based on the DrugVirus dataset, we have screened several machine learning–based models belonging to different types and multiple deep learning models. Then we ensembled four matrix completion models as the optimal combination according to their performance indicators. (3) We have demonstrated reliable results for the EMC-Voting model through multiple cross-validation experiments and case studies. It can be found that our model has good predictive performance and can effectively assist clinicians in selecting possible therapeutic drugs.

Based on the characteristics of the virus-drug database, with sparse, no negative samples and extremely unbalanced data, we screened several matrix completion models and ensembled four to predict drugs that may have therapeutic effects on SARS-COV-2, so it can assist clinical doctors to identify effective drugs at an urgent time. Furthermore, based on preclinical and clinical studies in which the database is being updated, the scope of BSAAs and their indications will be expanded in the future, and drug combinations for the treatment of emerging and re-emerging viral infections and co-infections will be identified (Chen et al., 2016; Ding et al., 2019).

## Data Availability Statement

The datasets presented in this study can be found in online repositories. The names of the repository/repositories and accession number(s) can be found below: https://github.com/vivian457/COVID-19_drug_virus_association.

## Author Contributions

WL, SW, and JX conceptualized the work and planned the procedure for experiments. JX collected the data. WL implemented all the experiments, analyzed the results, and drafted the manuscript. All authors have read and supported the final edition.

## Conflict of Interest

The authors declare that the research was conducted in the absence of any commercial or financial relationships that could be construed as a potential conflict of interest.

## Footnotes

1 https://DrugVirus.info/

2 https://string-db.org

3 https://www.kegg.jp/

## References

[B1] AlJame M. Ahmad I. Imtiaz A. Mohammed A. (2020). Ensemble learning model for diagnosing COVID-19 from routine blood tests. *Inform. Med. Unlocked* 21:100449. 10.1016/j.imu.2020.100449 33102686 PMC7572278

[B2] Andersen P. I. Ianevski A. Lysvand H. Vitkauskiene A. Oksenych V. Bjørås M. (2020). Discovery and development of safe-in-man broad-spectrum antiviral agents. *Int. J. Infect. Dis.* 93 268–276. 10.1016/j.ijid.2020.02.018 32081774 PMC7128205

[B3] Cai J. Luo J. Wang S. Yang S. (2018). Feature selection in machine learning: a new perspective. *Neurocomputing* 300 70–79. 10.1016/j.neucom.2017.11.077

[B4] Cai Q. Yang M. Liu D. Chen J. Shu D. Xia J. (2020). Experimental treatment with favipiravir for COVID-19: an open-label control study. *Engineering* 6 1192–1198. 10.1016/j.eng.2020.03.007 32346491 PMC7185795

[B5] Calderón J. M. Figueroa Flores M. D. R. Coria L. P. Briones Garduño J. C. Figueroa J. M. Vargas Contreras M. J. (2020). Nitazoxanide against COVID-19 in three explorative scenarios. *J. Infect. Dev. Ctries.* 14 982–986. 10.3855/JIDC.13274 33031085

[B6] Candès E. J. Recht B. (2009). Exact matrix completion via convex optimization. *Found. Comput. Math.* 9:717. 10.1007/s10208-009-9045-5

[B7] Chen L. Xu J. Li S. C. (2019). DeepMF: deciphering the latent patterns in omics profiles with a deep learning method. *BMC Bioinformatics* 20:648. 10.1186/s12859-019-3291-6 31881818 PMC6933662

[B8] Chen X. Guan N. Sun Y. Li J. Qu J. (2018a). MicroRNA-small molecule association identification: from experimental results to computational models. *Brief. Bioinformatics* 21 1–15. 10.1093/bib/bby098 30325405

[B9] Chen X. Liu M. X. Yan G. Y. (2012). Drug-target interaction prediction by random walk on the heterogeneous network. *Mol. Biosyst.* 8 1970–1978. 10.1039/c2mb00002d 22538619

[B10] Chen X. Ren B. Chen M. Wang Q. Zhang L. Yan G. (2016). NLLSS: predicting synergistic drug combinations based on semi-supervised learning. *PLoS Comput. Biol.* 12:e1004975. 10.1371/journal.pcbi.1004975 27415801 PMC4945015

[B11] Chen X. Sun L. G. Zhao Y. (2021). NCMCMDA: miRNA-disease association prediction through neighborhood constraint matrix completion. *Brief. Bioinform.* 22 485–496. 10.1093/bib/bbz159 31927572

[B12] Chen X. Wang L. Qu J. Guan N. N. Li J. Q. (2018b). Predicting miRNA-disease association based on inductive matrix completion. *Bioinformatics* 34 4256–4265. 10.1093/bioinformatics/bty503 29939227

[B13] Chen Y. Ma T. Yang X. Wang J. Song B. Zeng X. (2021). MUFFIN: multi-scale feature fusion for drug–drug interaction prediction. *Bioinformatics* 2021:btab169. 10.1093/bioinformatics/btab169 33720331

[B14] Choy K.-T. T. Wong A. Y.-L. L. Kaewpreedee P. Sia S. F. Chen D. Hui K. P. Y. (2020). Remdesivir, lopinavir, emetine, and homoharringtonine inhibit SARS-CoV-2 replication in vitro. *Antiviral Res.* 178:104786. 10.1016/j.antiviral.2020.104786 32251767 PMC7127386

[B15] Deng Y. Xu X. Qiu Y. Xia J. Zhang W. Liu S. (2020). A multimodal deep learning framework for predicting drug-drug interaction events. *Bioinformatics* 36 4316–4322. 10.1093/bioinformatics/btaa501 32407508

[B16] Ding P. Yin R. Luo J. Kwoh C. K. (2019). Ensemble prediction of synergistic drug combinations incorporating biological, chemical, pharmacological, and network knowledge. *IEEE J. Biomed. Health Inform.* 23 1336–1345. 10.1109/JBHI.2018.2852274 29994408

[B17] Driouich J. S. Cochin M. Lingas G. Moureau G. Touret F. Petit P. R. (2021). Favipiravir antiviral efficacy against SARS-CoV-2 in a hamster model. *Nat. Commun.* 12:1735. 10.1038/s41467-021-21992-w 33741945 PMC7979801

[B18] Ezzat A. Zhao P. Wu M. Li X. L. Kwoh C. K. (2017). “Drug-target interaction prediction with graph regularized matrix factorization,” in *Proceedings of the IEEE/ACM Transactions on Computational Biology and Bioinformatics*, Vol. 14 (Piscataway, NJ: IEEE), 646–656. 10.1109/TCBB.2016.2530062 26890921

[B19] Fan J. Cheng J. (2018). Matrix completion by deep matrix factorization. *Neural Netw.* 98 34–41. 10.1016/j.neunet.2017.10.007 29154225

[B20] Fan J. Chow T. (2017). Deep learning based matrix completion. *Neurocomputing* 266 540–549. 10.1016/j.neucom.2017.05.074

[B21] Gao Y. Chen L. Chi J. Zeng S. Feng X. Li H. (2021). Development and validation of an online model to predict critical COVID-19 with immune-inflammatory parameters. *J. Intensive Care* 9:19. 10.1186/s40560-021-00531-1 33602326 PMC7891473

[B22] Gu Q. Zhou J. Ding C. (2010). “Collaborative filtering: weighted nonnegative matrix factorization incorporating user and item graphs,” in *Proceedings of the 10th SIAM International Conference on Data Mining SDM 2010* Columbus, OH, 199–210. 10.1137/1.9781611972801.18

[B23] Han Y. Duan X. Yang L. Nilsson-Payant B. E. Wang P. Duan F. (2021). Identification of SARS-CoV-2 inhibitors using lung and colonic organoids. *Nature* 589 270–275. 10.1038/s41586-020-2901-9 33116299 PMC8034380

[B24] Hastie T. Mazumder R. Lee J. D. Zadeh R. (2015). Matrix completion and low-rank SVD via fast alternating least squares. *J. Mach. Learn. Res.* 16 3367–3402. 31130828 PMC6530939

[B25] Hattori M. Tanaka N. Kanehisa M. Goto S. (2010). SIMCOMP/SUBCOMP: chemical structure search servers for network analyses. *Nucleic Acids Res.* 38 W652–W656. 10.1093/nar/gkq367 20460463 PMC2896122

[B26] He B. S. Yang H. Wang S. L. (2000). Alternating direction method with self-adaptive penalty parameters for monotone variational inequalities. *J. Optim. Theory Appl.* 106 337–356. 10.1023/A:1004603514434

[B27] Kamneva O. K. (2017). Genome composition and phylogeny of microbes predict their co-occurrence in the environment. *PLoS Comput. Biol.* 13:e1005366. 10.1371/journal.pcbi.1005366 28152007 PMC5313232

[B28] Li J. Zheng S. Chen B. Butte A. J. Swamidass S. J. Lu Z. (2016). A survey of current trends in computational drug repositioning. *Brief. Bioinform.* 17 2–12. 10.1093/bib/bbv020 25832646 PMC4719067

[B29] Li W. Wang S. Xu J. Mao G. Tian G. Yang J. (2019). Inferring latent disease-lncRNA associations by faster matrix completion on a heterogeneous network. *Front. Genet.* 10:769. 10.3389/fgene.2019.00769 31572428 PMC6749816

[B30] Li X. Geng M. Peng Y. Meng L. Lu S. (2020). Molecular immune pathogenesis and diagnosis of COVID-19. *J. Pharm. Anal.* 10 102–108. 10.1016/j.jpha.2020.03.001 32282863 PMC7104082

[B31] Liu H. Ren G. Chen H. Liu Q. Yang Y. Zhao Q. (2020). Predicting lncRNA–miRNA interactions based on logistic matrix factorization with neighborhood regularized. *Knowl. Based Syst.* 191:105261. 10.1016/j.knosys.2019.105261

[B32] Long Y. Luo J. (2020). Association mining to identify microbe drug interactions based on heterogeneous network embedding representation. *IEEE J. Biomed. Health Informatics* 2194 1–1. 10.1109/jbhi.2020.2998906 32750918

[B33] Long Y. Wu M. Kwoh C. K. Luo J. Li X. (2020a). Predicting human microbe-drug associations via graph convolutional network with conditional random field. *Bioinformatics* 36 4918–4927. 10.1093/bioinformatics/btaa598 32597948 PMC7559035

[B34] Long Y. Wu M. Liu Y. Kwoh C. K. Luo J. Li X. (2020b). Ensembling graph attention networks for human microbe-drug association prediction. *Bioinformatics* 36 I779–I786. 10.1093/bioinformatics/btaa891 33381844

[B35] Luo J. Ding P. Liang C. Chen X. (2018). Semi-supervised prediction of human miRNA-disease association based on graph regularization framework in heterogeneous networks. *Neurocomputing* 294 29–38. 10.1016/j.neucom.2018.03.003

[B36] Luo J. Long Y. (2020). NTSHMDA: prediction of human microbe-disease association based on random walk by integrating network topological similarity. *IEEE/ACM Trans. Comput. Biol. Bioinform.* 17 1341–1351. 10.1109/TCBB.2018.2883041 30489271

[B37] Luo J. Xiao Q. (2017). A novel approach for predicting microRNA-disease associations by unbalanced bi-random walk on heterogeneous network. *J. Biomed. Inform.* 66 194–203. 10.1016/j.jbi.2017.01.008 28104458

[B38] Mani J. S. Johnson J. B. Steel J. C. Broszczak D. A. Neilsen P. M. Walsh K. B. (2020). Natural product-derived phytochemicals as potential agents against coronaviruses: a review. *Virus Res.* 284:197989. 10.1016/j.virusres.2020.197989 32360300 PMC7190535

[B39] NCT04471662 (2020). *Nelfinavir and Favipiravir Combination in Newly Diagnosed COVID19 Egyptian Patients.* Available online at: https://clinicaltrials.gov/show/NCT04471662 (accessed July 31, 2020).

[B40] NCT04575038 (2020). *CRISIS2: a Phase 2 Study Assessing the Safety and Antiviral Activity of Brequinar in Non-Hospitalized Patients with COVID-19.* Available online at: https://clinicaltrials.gov/show/NCT04575038 (accessed October 31, 2020).

[B41] Peng L. H. Sun C. N. Guan N. N. Li J. Q. Chen X. (2018). HNMDA: heterogeneous network-based miRNA–disease association prediction. *Mol. Genet. Genomics* 293 983–995. 10.1007/s00438-018-1438-1 29687157

[B42] Ramlatchan A. Yang M. Liu Q. Li M. Wang J. Li Y. (2018). A survey of matrix completion methods for recommendation systems. *Big Data Min. Anal.* 1 308–323. 10.26599/bdma.2018.9020008

[B43] Sareen K. Bose R. Singh R. Boddu L. (2020). Treatment of COVID-19. *Prax. Undergraduate Med. Res. J.* 3 56–64.

[B44] Varghese F. S. van Woudenbergh E. Overheul G. J. Eleveld M. J. Kurver L. van Heerbeek N. (2021). Berberine and obatoclax inhibit sars-cov-2 replication in primary human nasal epithelial cells in vitro. *Viruses* 13:282. 10.3390/v13020282 33670363 PMC7918080

[B45] Wang B. Ge L. S. Huang D. S. Wong H. S. (2007a). “Prediction of protein-protein interacting sites by combining SVM algorithm with Bayesian method,” in *Proceedings of the 3rd International Conference on Natural Computation, ICNC 2007*, Haikou. 10.1109/ICNC.2007.562

[B46] Wang B. Ge L. S. Jia W. Y. Liu L. Chen F. C. (2007b). “Prediction of protein interactions by combining genetic algorithm with SVM method,” in *Proceedings of the 2007 IEEE Congress on Evolutionary Computation CEC 2007*, Singapore, 320–325. 10.1109/CEC.2007.4424488

[B47] Wang Y. Deng G. Zeng N. Song X. Zhuang Y. (2019). Drug-disease association prediction based on neighborhood information aggregation in neural networks. *IEEE Access* 7 50581–50587. 10.1109/ACCESS.2019.2907522

[B48] Weiss A. Touret F. Baronti C. Gilles M. Hoen B. Nougairède A. (2021). Niclosamide shows strong antiviral activity in a human airway model of SARS-CoV-2 infection and 2 a conserved potency against the UK B.1.1.7 and SA B.1.351 variant 3. *bioRxiv* [Preprint]. 10.1101/2021.04.26.441457 bioRxiv:2021.04.26.441457 PMC8639074 34855904

[B49] Weston S. Coleman C. M. Sisk J. M. Haupt R. Logue J. Matthews K. (2020). Broad anti-coronaviral activity of FDA approved drugs against SARS-CoV-2 in vitro and SARS-CoV in vivo. *bioRxiv* [Preprint]. 10.1101/2020.03.25.008482 PMC7565640 32817221

[B50] Xu J. Shi P. Y. Li H. Zhou J. (2020). Broad spectrum antiviral agent niclosamide and its therapeutic potential. *ACS Infect. Dis.* 6 909–915. 10.1021/acsinfecdis.0c00052 32125140 PMC7098069

[B51] Yang M. Huang L. Xu Y. Lu C. Wang J. (2021). Heterogeneous graph inference with matrix completion for computational drug repositioning. *Bioinformatics* 36 5456–5464. 10.1093/bioinformatics/btaa1024 33331887

[B52] Yang M. Luo H. Li Y. Wang J. (2019). Drug repositioning based on bounded nuclear norm regularization. *Bioinformatics* 35 i455–i463. 10.1093/bioinformatics/btz331 31510658 PMC6612853

[B53] Yu Z. Huang F. Zhao X. Xiao W. Zhang W. (2020). Predicting drug–disease associations through layer attention graph convolutional network. *Brief. Bioinform.* 2020:bbaa243. 10.1093/bib/bbaa243 33078832

[B54] Zeng X. Zhong Y. Lin W. Zou Q. (2019). Predicting disease-associated circular RNAs using deep forests combined with positive-unlabeled learning methods. *Brief. Bioinform.* 21 1425–1436. 10.1093/bib/bbz080 31612203

[B55] Zeng X. Zhu S. Hou Y. Zhang P. Li L. Li J. (2020). Network-based prediction of drug-target interactions using an arbitrary-order proximity embedded deep forest. *Bioinformatics* 36 2805–2812. 10.1093/bioinformatics/btaa010 31971579 PMC7203727

[B56] Zhang L. Liu T. Chen H. Zhao Q. Liu H. (2021). Predicting lncRNA–miRNA interactions based on interactome network and graphlet interaction. *Genomics* 113 874–880. 10.1016/j.ygeno.2021.02.002 33588070

[B57] Zhang W. Jing K. Huang F. Chen Y. Li B. Li J. (2019a). SFLLN: a sparse feature learning ensemble method with linear neighborhood regularization for predicting drug–drug interactions. *Inform. Sci.* 497 189–201. 10.1016/j.ins.2019.05.017

[B58] Zhang W. Li Z. Guo W. Yang W. Huang F. (2019b). A fast linear neighborhood similarity-based network link inference method to predict microRNA-disease associations. *IEEE/ACM Trans. Comput. Biol. Bioinform.* 18 405–415. 10.1109/tcbb.2019.2931546 31369383

[B59] Zhang W. Lu X. Yang W. Huang F. Wang B. Wang A. (2019c). “HNGRNMF: heterogeneous network-based graph regularized nonnegative matrix factorization for predicting events of microbe-disease associations,” in *Proceedings of the 2018 IEEE International Conference on Bioinformatics and Biomedicine BIBM 2018*, Madrid, 803–807. 10.1109/BIBM.2018.8621085

[B60] Zhang W. Yue X. Huang F. Liu R. Chen Y. Ruan C. (2018). Predicting drug-disease associations and their therapeutic function based on the drug-disease association bipartite network. *Methods* 145 51–59. 10.1016/j.ymeth.2018.06.001 29879508

[B61] Zhao Q. Yu H. Ming Z. Hu H. Ren G. Liu H. (2018). The bipartite network projection-recommended algorithm for predicting long non-coding RNA-protein interactions. *Mol. Ther. Acids* 13 464–471. 10.1016/j.omtn.2018.09.020 30388620 PMC6205413

[B62] Zhao Y. Chen X. Yin J. (2019). Adaptive boosting-based computational model for predicting potential miRNA-disease associations. *Bioinformatics* 35 4730–4738. 10.1093/bioinformatics/btz297 31038664

